# The Complete Plastome Sequences of Eleven *Capsicum* Genotypes: Insights into DNA Variation and Molecular Evolution

**DOI:** 10.3390/genes9100503

**Published:** 2018-10-17

**Authors:** Nunzio D’Agostino, Rachele Tamburino, Concita Cantarella, Valentina De Carluccio, Lorenza Sannino, Salvatore Cozzolino, Teodoro Cardi, Nunzia Scotti

**Affiliations:** 1 CREA Research Centre for Vegetable and Ornamental Crops, Via dei Cavalleggeri 25, 84098 Pontecagnano Faiano (SA), Italy; concita.cantarella@gmail.com (C.C.); valentina.decarluccio@gmail.com (V.D.C.); teodoro.cardi@crea.gov.it (T.C.); 2 CNR-IBBR, National Research Council of Italy, Institute of Biosciences and BioResources, Via Università 133, 80055 Portici (NA), Italy; rachele.tamburino@gmail.com (R.T.); lorenza.sannino@ibbr.cnr.it (L.S.); 3 Department of Biology, University of Naples Federico II, Via Cinthia, 80126 Naples, Italy; cozzolin@unina.it

**Keywords:** chloroplast genome, pepper, next-generation sequencing, sequence variability, single-nucleotide polymorphism, simple sequence repeats, microsatellites, perfect tandem repeats, molecular markers

## Abstract

Members of the genus *Capsicum* are of great economic importance, including both wild forms and cultivars of peppers and chilies. The high number of potentially informative characteristics that can be identified through next-generation sequencing technologies gave a huge boost to evolutionary and comparative genomic research in higher plants. Here, we determined the complete nucleotide sequences of the plastomes of eight *Capsicum* species (eleven genotypes), representing the three main taxonomic groups in the genus and estimated molecular diversity. Comparative analyses highlighted a wide spectrum of variation, ranging from point mutations to small/medium size insertions/deletions (InDels), with *accD*, *ndhB*, *rpl20*, *ycf1*, and *ycf2* being the most variable genes. The global pattern of sequence variation is consistent with the phylogenetic signal. Maximum-likelihood tree estimation revealed that *Capsicum chacoense* is sister to the *baccatum* complex. Divergence and positive selection analyses unveiled that protein-coding genes were generally well conserved, but we identified 25 positive signatures distributed in six genes involved in different essential plastid functions, suggesting positive selection during evolution of *Capsicum* plastomes. Finally, the identified sequence variation allowed us to develop simple PCR-based markers useful in future work to discriminate species belonging to different *Capsicum* complexes.

## 1. Introduction

In recent decades, plastid DNA (cpDNA) markers were used either to infer species-level phylogenetic and phylogeographic relationships in plants or to identify species via barcoding approaches [1,2,3]. Although cpDNA sequence divergence is often unable to provide adequate resolution of genetic differences at the intra-specific level because of its slow evolutionary rate, chloroplast DNA-based molecular markers, such as microsatellites and tandem repeats, are widely exploited to reveal inter-specific variation [4,5,6]. The progress of high-throughput sequencing technologies and the relatively simple assembling process of cpDNA gave a huge boost to genomic and phylogenetic studies [5,6,7,8]. As chloroplast genomes are characterized by a high number of potentially informative nucleotide sites, they became an efficient and cost-effective option for evolutionary and comparative genomic research in higher plants [9,10,11,12,13,14].

The genus *Capsicum* (Solanaceae), native to South and Central America and the southern United States of America (USA), includes sweet (peppers) and hot (chillies) cultivars of great economic importance that are cultivated and consumed around the world as vegetables and spices rich in vitamins A and C [15,16,17]. *Capsicum*-specific starch fossils, found at seven sites from the Bahamas to southern Peru, dating 6000 years before first contact with Europeans, clearly demonstrate that members of the genus *Capsicum* were extensively cultivated initially in the Americas and, after Columbus, were dispersed around the World [18]. *Capsicum* species exhibit flowers with stellate or rotate corollas characterized by diverse patterns of pigmentation and fleshy berries, which differ in shape, size, and color. In addition, plants of the genus *Capsicum* show an entire cup-shaped calyx, a unique trait among Solanaceae flowers and only shared with flowers of the genus *Lycianthes* [15].

Taxonomic studies of the genus, based on morphological, cytogenetic, biochemical, and ethnobotanical data, grouped *Capsicum* species into three main complexes: *annuum* (C_A_), *baccatum* (C_B_), and *pubescens* (C_P_). The C_A_ complex includes wild and domesticated species of *C. annuum*, *Capsicum chinense*, *Capsicum frutescens*, and *Capsicum galapagoense*; the C_B_ complex contains *C. baccatum*, *Capsicum praetermissum*, and *Capsicum tovarii*, whereas the C_P_ complex comprises *C. pubescens*, *Capsicum eximium*, and *Capsicum cardenasii* [19,20]. *Capsicum chacoense* is considered a bridge species that could be included in either the C_A_ or C_B_ complex [19]. Although this classification is commonly accepted, establishing the genetic relationships within and between *Capsicum* species is still debated. Many studies were performed using different nuclear and plastid molecular markers to gain a better understanding of the genetic relationships within *Capsicum* and to assess genetic diversity in populations or core collections [5,15,16,21,22,23].

Next-generation sequencing (NGS) technologies provided a significant advancement in high-throughput data generation; however, the simultaneous analysis of a large number of genotypes is still challenging. Indeed, the whole chloroplast genome sequences of few *Capsicum* species have been released so far into the public domain [24,25,26,27,28,29,30]. The availability of a larger set of complete plastomes would allow for a better understanding of nuclear and cytoplasmic genome co-evolution, and would favor the development of more powerful methods for taxonomic barcoding and phylogenetic studies, as well as of novel biotechnological approaches for breeding purposes [31,32,33].

Here, we determined the complete nucleotide sequences of plastomes of eleven genotypes belonging to eight *Capsicum* species representing the three main taxonomic complexes, and performed a genome-wide analysis of molecular diversity among *Capsicum* plastomes. In addition to the assembly and annotation of plastomes, our aims were to (i) measure global patterns of sequence variations and establish the relationships among sequenced species; (ii) evaluate the extent of sequence similarity between plastomes; (iii) investigate any significant characteristics suggesting plastome rearrangements in *Capsicum*; (iv) derive estimates for molecular evolution of plastid protein-coding genes within *Capsicum*; (vi) and identify divergent regions suitable for the development of simple PCR-based molecular markers as a baseline to discriminate among *Capsicum* species.

## 2. Materials and Methods

### 2.1. Plant Material

A panel of eleven genotypes representing three complexes of the genus *Capsicum* was sampled for chloroplast isolation, cpDNA extraction, and sequencing. For the C_A_ complex, we sampled three *C. annuum* genotypes (ann1, ann2, and ann3) and one genotype each for the species *C. chinense* (chi), *C. frutescens* (fru) and *C. galapagoense* (gal). For the C_B_ complex, we sampled *C. baccatum* subsp. *baccatum* (bac.b), *C. baccatum* subsp. *pendulum* (bac.p), and *C. praetermissum* (pra). Finally, we also included a genotype from the C_P_ complex, namely *C. pubescens* (pub), and a *C. chacoense* genotype (cha) that, depending on the classification schemes, is included in either C_A_ or in C_B_ (Table 1).

Seeds that were provided by the Centre for Genetic Resources (Wageningen, The Netherlands) and Institut für Pflanzengenetik und Kulturpflanzenforschung (IPK, Gatersleben, Germany) were germinated in the presence of 3 mM gibberellic acid. After germination, seedlings were transferred into pots and cultivated in a greenhouse under controlled conditions.

### 2.2. Chloroplast Isolation and DNA Extraction

Plants were kept in the dark for 48 h before harvesting to reduce starch contamination. A pool of about 15–25 g of fresh leaves collected from different individuals were used for chloroplast isolation with discontinuous sucrose gradients according to Kemble [34]. Purified chloroplasts were lysed with a detergent and the resulting cpDNA was dissociated from the proteins using proteinase K and phenol/chloroform treatments following the procedure described in Scotti et al. [35].

### 2.3. Chloroplast DNA Sequencing and Genome Assembly

Genomic libraries of *C. baccatum* subsp. *baccatum*, *C. frutescens*, *C. praetermissum*, and *C. pubescens* were sequenced on an Illumina HiSeq 2500 using the Illumina TruSeq DNA (Illumina, San Diego, CA, USA) sample preparation kit with 2 × 101 paired-end runs. The remaining plastomes were sequenced on an Illumina MiSeq platform with 2 × 251 paired-end runs after library preparation with an Illumina Nextera XT sample preparation kit.

High-quality reads were aligned onto the reference *C. annuum* cpDNA (NC_018552.1) with the BWA software [36] (version 0.7.12; Heng Li and Richard Durbin, Wellcome Trust Sanger Institute, Cambridge, UK). The Picard software (version 1.131; Broad Institute of MIT, Cambridge, MA, USA) was used to collect metrics (mean and standard deviation) on insert size distribution of each paired-end library. The Velvet software (version 1.2.10; Daniel R. Zerbino, Wellcome Trust Sanger Institute, Cambridge, UK) [37] was used for de novo assembly with the following settings: -ins_length XX, -ins_length_sd YY, scaffolding yes, and -exp_cov 100. Values XX and YY were obtained from the Picard analysis. Kmer length was set to 95 for all samples with the exception of *C. chinense* chi (kmer = 121) and pra *C. praetermissum* (kmer = 89).

### 2.4. Genome Annotation and Analysis of Nucleotide Variability

Genome annotation was carried out using the web tool DOGMA [38]. Gene structures were manually curated using *Nicotiana tabacum* (NC_001879.2) and *Solanum lycopersicum* (NC_007898.3) structural annotations as references. The circular *C. pubescens* plastome map was drawn using the online webtool OGDRAW—Draw Organelle Genome Maps [39]. Newly assembled chloroplast genomes and the plastome of *Capsicum lycianthoides* (NC_026551) were subjected to multiple alignment using the ClustalW program [40].

Genetic variability among pepper cpDNAs was investigated using different bioinformatic tools. Single-nucleotide variants (SNVs) were identified using the SNP-sites software [41] (Wellcome Trust Sanger Institute, Cambridge, UK). Such a tool extracted single-nucleotide polymorphisms (SNPs) from a multiple-sequence alignment using the cpDNA of *C. lycianthoides* as the reference sequence. Microsatellites (simple sequence repeats (SSRs)) were identified running the MIcroSAtellite (MISA) identification tool (http://pgrc.ipk-gatersleben.de/misa/) using the unit_size/min_repeats parameters as follows: 1/8, 2/6, 3/5, 4/5, 5/5, 6/5. Tandem repeats were identified using the Tandem Repeat Finder web tool accessible at https://tandem.bu.edu/trf/trf.basic.submit.html. Only perfect repeats were considered for downstream analysis. To visualize the overall differences among plastomes, we built pairwise alignments among eleven *Capsicum* plastomes by running mVISTA (Stanford University, Stanford, CA, USA) in LAGAN (Limited Area Global Alignment of Nucleotides) mode [42] and using the annotation of *C. lycianthoides* (NC_026551) as a reference. Aligned plastomes were also used to perform sliding window analysis using the DnaSP software [43] (University of Barcelona, Barcelona, Spain).

Seven regions, namely *trnH-psbA*, *matK*, *rps16*, *trnL* intron, *atpB-rbcL*, *rbcL*, and *ndhF*, that are traditionally used in previous phylogenetic studies based on Sanger sequencing methods [15,16,18,19,20] were used to infer a maximum-likelihood (ML) phylogenetic tree. The regions were extracted from each plastome using a custom perl script; then, a concatemer per genotype was generated, and a multiple-sequence alignment was built and manually inspected using the Bioedit software (Tom Hall, Ibis Therapeutics, Carlsbad, ON, Canada). An ML tree with 10,000 rapid bootstrap inferences, a generalized time reversible (GTR) substitution matrix and Gamma model of rate heterogeneity was inferred using the RAxML (The Exelixis Lab 2013, Scientific Computing Group, Heidelberg, Germany) program [44]. The RAxML results were visualized with the FigTree software, v.1.4.2 (http://tree.bio.ed.ac.uk/software/figtree/). The same approach was used to infer a second tree based on the alignment of complete plastid sequences of the same genotypes.

*Capsicum* chloroplast genomes released into the public domain with accession numbers NC_028007.1/KR078312.1 (*C. frutescens*), NC_033525.1/KX913218.1 (*C. chacoense*), NC_030543.1/KU041709.1 (*C. chinense*), NC_033524.1/KX913216.1 (*C. galapagoense*), NC_018552.1/JX270811.1 (*C. annuum*), KR078313.1 (*C. annuum*), and KR078314.1 (*C. baccatum* var. *baccatum*) were downloaded from GenBank.

Pairwise global alignments between already publically available chloroplast sequences and plastomes that were sequenced and assembled in this study were performed using the European Molecular Biology Open Software Suite (EMBOSS) Stretcher tool. SNVs were identified using SNP-sites [41], while insertions/deletions (InDels) were manually scored.

### 2.5. Molecular Evolution Analysis on Protein-Coding Genes

The coding sequences of the 79 protein-coding genes present in all *Capiscum* plastomes and in *C. lycianthoides* (NC_026551) were extracted and fed into the Selecton web server [45] (http://selecton.tau.ac.il/) in order to investigate amino-acid sites under positive selection. The evolutionary model M8a (ωs = 1) was used. We considered a site under positive selection if the lower bound was >1 and the *p*-value was <0.01.

### 2.6. Primer Design and PCR Amplification

Primers for the development of *ccsA-ndhD* (forward (F): ACACATAGAAATTTGCGGGGTGC; reverse (R): TCGATGGCTTCCCTTGCATTACCA) and *trnL-trnF* (F: ATCGAAGAAATTCCCCGGCT; R: GCGCACATTACTTAGACGGGTT) molecular markers were designed from assembled plastomes by using the MacVector software (MacVector Inc., Apex, NC, USA). PCR amplifications were carried out, using Taq DNA polymerase according to the manufacturer’s instructions (Invitrogen, Paisley, UK), on 25 ng of total DNA or cpDNA of the following genotypes: *C. baccatum* subsp. *baccatum* (bac.b, Table 1), *C. baccatum* subsp. *pendulum* (bac.p, Table 1; bac.p2, CGN17015; bac.p3, CGN22181; bac.p4, CGN17174), *C. praetermissum* (pra, Table 1), *C. pubescens* (pub, Table 1; pub2, CGN22796; pub3, CAP1486), *C. chacoense* (cha, Table 1; cha2, CAP1445; cha3, CAP499; cha4, CAP501), *C. annuum* (ann2, Table 1; ann4, CGN17175; ann5, CGN21490; ann6, CGN24355; ann7, CGN23249), *C. chinense* (chi2, CGN17220; chi3, CGN23565; chi4, CGN17219), and *C. frutescens* (fru2, CGN22792; fru3, RCAT077650; fru4, CGN21546)*.* The reaction conditions for all amplifications were as follows: denaturation at 94 °C for 3 min, then 30 cycles (94 °C, 30 s; annealing temperature, 30 s; 72 °C, 1 min/kb), followed by 5 min final extension at 72 °C.

## 3. Results

### 3.1. Chloroplast Genome Size and Organization

Sequencing of the eleven *Capsicum* genotypes produced 5,634,814–404,910,769 base pairs (bp) of high-quality plastid reads with per-base mean coverage ranging from 26 to 2581. A combination of de novo and reference-guided assembly with the *C. annuum* chloroplast genome (NC_018552.1) as a reference was used to obtain the complete plastome for all genotypes. Genome sizes ranged from 156,836 bp in *C. frutescens* to 157,390 in *C. pubescens* (Table 1).

**Figure 1:**
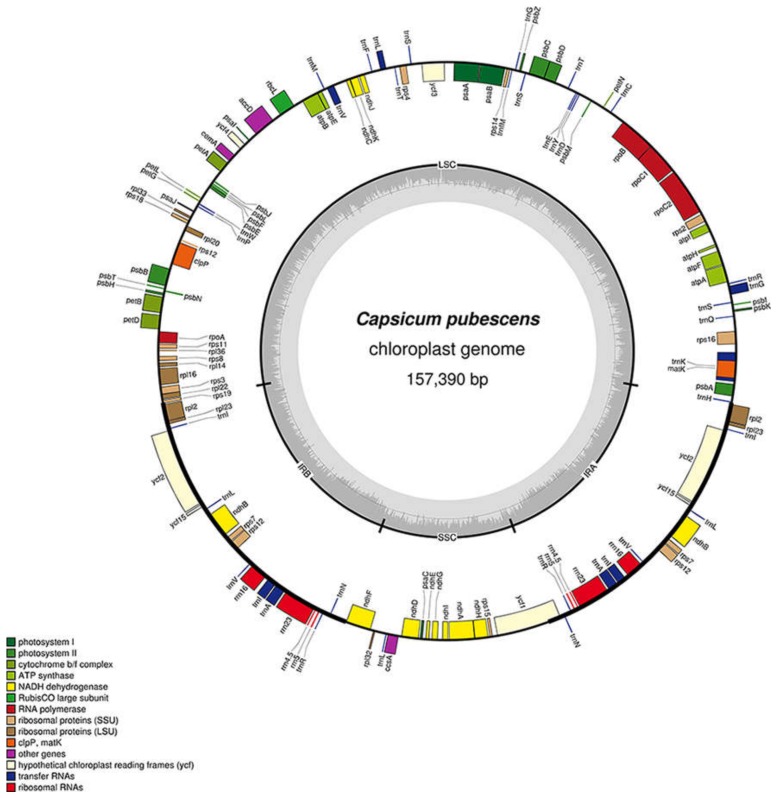
Map of the *Capsicum pubescens* chloroplast genome. Genes inside of the outer circle are transcribed in the clockwise direction, while those outside are transcribed in the counterclockwise direction. Different color codes represent genes belonging to various functional groups. The circle inside GC content graph marks the 50% threshold. The inverted repeat, large single-copy, and small single-copy regions are denoted by IR, LSC, and SSC, respectively.

As expected, all *Capsicum* genotypes exhibited the typical quadripartite structure of angiosperms, including a pair of inverted repeats (IRs), ranging from 25,751 bp to 25,910 bp in size, separated by two single-copy regions, a large single copy (LSC, 87,288 bp min–87,688 bp max) and a small single copy (SSC, 17,860 bp min–17,973 bp max). A slight variation in guanine/cytosine (GC) content among genotypes was observed (Table 1). Each of the eleven plastomes, similar to other Solanaceae, contains 113 genes, including 79 protein-coding, four ribosomal RNA, and 30 transfer RNA (tRNA) genes. Seventeen genes, located in IR regions, were duplicated (Figure 1).

A detailed view of the IR–SSC/LSC junctions of the plastomes under investigation is provided in the Supplementary Materials (Figure S1). In all genotypes, the LSC/IRb and SSC/IRa junctions are in the *rps*19 and *ycf*1 genes, respectively, while the IRb/SSC and IRa/LSC ones are in the intergenic *trnN–GUU/ndhF* and *rpl2/trnH–GUG* regions. The junction position, however, slightly varies among different genotypes.

The eleven plastomes were deposited in GenBank under accession numbers: MH559320–MH559330.

### 3.2. Sequence Variation within Capsicum Genotypes

**Figure 2:**
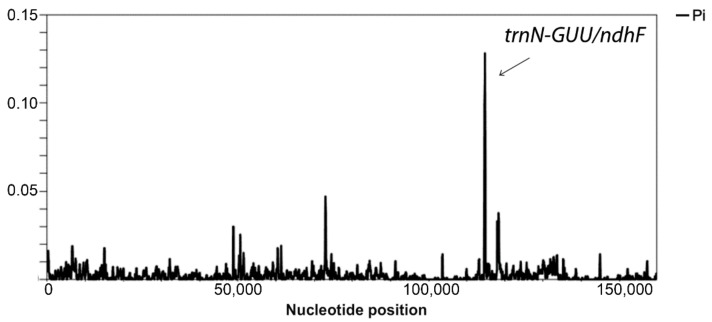
Sliding window analysis of the multiple plastome sequence alignment within the *Capsicum* genus. The region with high nucleotide variability (Pi > 0.05), corresponding to the IR/SSC junction, is indicated. Window length = 200 base pairs (bp); step size = 50 bp.

All comparative analyses across pepper genotypes were carried out using *C. lycianthoides* (NC_026551) as a reference genome. Sliding window analysis of the multiple-sequence alignment including the eleven *Capsicum* plastomes and *C. lycianthoides* showed high sequence similarity and indicated the *trnN–GUU/ndhF* intergenic region as a polymorphic hotspot (Figure 2).

Similarly, VISTA-based identity plots revealed moderate sequence divergences among the genotypes under investigation. Indeed, nucleotide differentiation mainly affects intergenic/non coding regions, as well as single-copy regions (Supplementary Figure S2). In comparison with *C. lycianthoides*, all pepper genotypes showed a large deletion (over 500 bp) in the intergenic region between *ndhF* and *rpl32* genes (below 50% identity).

Variations SNPs, tandem repeats (TRs), and SSRs were assessed among the sequenced *Capsicum* genotypes. A range of 1152–1271 SNPs was detected among the eleven plastomes. The distribution of these variations in different regions was slight different among species and well conserved within genotypes belonging to the C_A_ complex (Supplementary Figure S3). In nine cases, two alternative alleles, compared to the reference, were discovered (Supplementary Materials Table S1). The C_B_ complex (including bac.b, bac.p, and pra) showed the highest SNP variations in intergenic (642–644), exon (497–500), and intron (125–130) regions, whereas the C_A_ complex (ann1, ann2, ann3, chi, fru, and gal) was characterized by 581–600, 462–470, and 105–111 SNPs in intergenic, exon, and intron regions, respectively. Intermediate values were detected for *C. chacoense* and *C. pubescens* (Supplementary Materials, Figure S3). Although the total number of detected SNPs seems almost equally distributed between intergenic and exon regions, normalization of SNP number per kb highlighted higher values in intergenic (13.99–15.51) compared to exon (6.09–6.58) regions (data not shown). SNP distribution within LSC, SSC, and IRb regions is also shown in the Supplementary Materials (Figure S3).

We identified 92 SSRs, of which 65 were polymorphic among the eleven *Capsicum* species, including mononucleotide, dinucleotide, trinucleotide, and tetranucleotide repeats (Supplementary Materials Figure S4A and Table S2). No pentanucleotide or hexanucleotide repeats were observed. The mononucleotide repeat (adenosine/thymine (A/T)) was the most common type of microsatellite in pepper plastomes, whereas the tetranucleotide unit, repeated four times, was typical of the C_A_ complex (Supplementary Materials Table S2). The distribution of SSRs showed that these loci were primarily located in intergenic regions and in the LSC, whilst the distribution in exon and intron regions and/or in the SSC and IRb was comparable (Supplementary Materials Figure S4B).

A total of 58 perfect tandem repeats (TRs) were identified, of which 51 are characterized by a period size of 9–30 bp, six have a period size ranging from 30 to 60 bp, and one is longer than 100 bp (Supplementary Materials Table S3). They are mostly located in intergenic regions (50), seven are in coding regions of *accD*, *rpl33*, *ycf2*, *ndhD*, and *ycf1*, and one was in the intron of the *rps16* gene. Most of them are located in the LSC region (Supplementary Materials Figure S5 and Table S3). Thirty-two of the 58 TRs are polymorphic within the eleven *Capsicum* plastomes, while three of them are located in the coding regions of *accD* and *ycf1*. A tandem repeat of 30 nucleotides located in the *ycf1* coding region is exclusive to the C_B_ complex.

**Figure 3:**
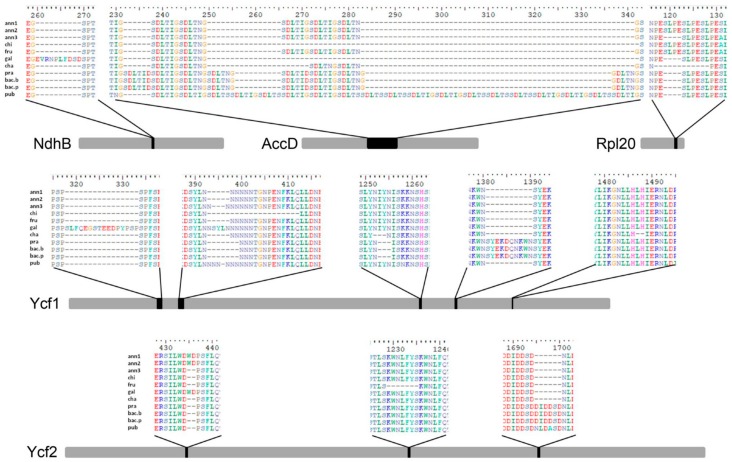
Schematic representation of the five most variable genes (*ndhB*, *accD*, *rpl20*, *ycf1* and *ycf2*) in the plastomes under investigation. Gray bars represent the multiple-sequence alignment (MSA) for each gene and are scaled according to the MSA length. Black boxes indicate highly variable regions in the MSA. Above each box, a snapshot of the MSA along with alignment positions is reported.

Among the annotated 79 protein-coding genes, 26 have perfectly conserved sequences and 48 have point mutations within the coding sequence, while five genes, namely *accD*, *ndhB*, *rpl20*, *ycf1*, and *ycf2* are the most variable. The latter differ in gene length because of several insertions/deletions, also evident at the amino-acid level (Figure 3).

In order to further evaluate within-species nucleotide variability, already publically available *Capsicum* chloroplast genomes belonging to the same species whose plastomes were sequenced in this work were downloaded from GenBank for comparative genomics. Species-specific pairwise global alignments showed nucleotide variability to be in the range of 0.1% to 0.3% (Supplementary Materials Table S4). Generally, nucleotide variability is in non-coding regions and affects A/T stretches, the number of tandem repeat units, and DNA low-complexity regions (data not shown).

### 3.3. Phylogenetic Reconstruction and Molecular Evolution

**Figure 4:**
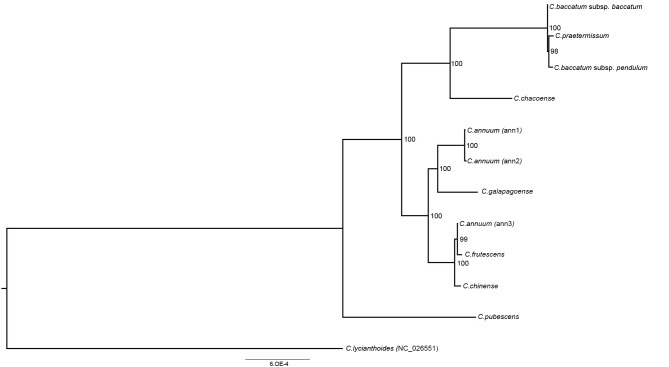
Phylogenetic tree of *Capsicum* genotypes. Phylogram of the best maximum-likelihood (ML) tree as determined using the RAxML software from the complete plastome dataset. Numbers associated with branches are ML bootstrap support values.

In order to reconstruct the phylogeny of *Capsicum* and to verify the evolutionary significance of SNP, SSR, and TR variation, a phylogenetic tree was inferred from plastid concatemers of seven regions (*trnH-psbA*, *matK*, *rps16*, *trnL* intron, *atpB-rbcL*, *rbcL*, and *ndhF*) from the eleven newly assembled pepper plastomes and *C. lycianthoides* (as an outgroup). The phylogenetic tree inferred from maximum-likelihood analysis has strong bootstrap supports for all nodes with the exception of the placement of *C. galapagoense* with respect to other species of the C_A_ complex, consisting of *C. annuum*, *C. frutescens*, and *C. chinense*. Nevertheless, the branch subtending the inclusion of *C. galapagoense* in the *C. annuum* clade is strongly supported. The *C. chacoense* genotype examined here is sister to the C_B_ complex with strong support. Finally, *C. pubescens* (a member of the C_P_ complex) is sister to both the *C. annuum* and *C. baccatum* lineages. The phylogenetic tree was compared with SNP variability in exon, intron, and intergenic regions (Supplementary Materials Figure S6), and with SSR and TR variation (Supplementary Materials Figure S7). In all cases, we found that the observed variability reflects the pattern of phylogenetic relationship resulting from the maximum-likelihood analysis. Based on this correspondence, we repeated the phylogenetic analysis using the alignment from the complete plastome sequences: the resulting ML tree (Figure 4) has the same topology as that based on concatemers of the seven plastid regions, but a stronger bootstrap supports all nodes.

**Figure 5:**
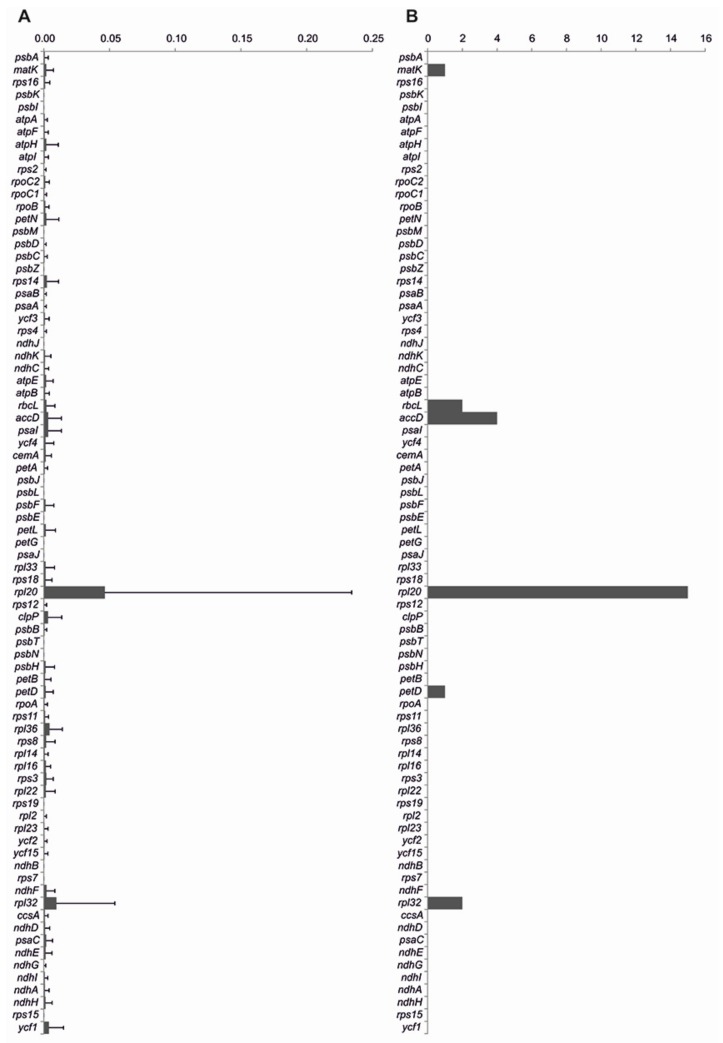
Results of molecular evolution analysis of plastid genes within the *Capsicum* genus. (**A**) Estimation of protein-coding gene divergence by the average branch length ± standard deviation for each gene tree; (**B**) number of putative sites under positive selection.

Gene divergence analysis, based on Selecton, showed that protein-coding genes are generally well conserved among *Capsicum* species. The most divergent genes are *rpl20* and *rpl32*, followed by *rpl36*, *clpP*, and *accD* (Figure 5A). This analysis also evidenced high divergent branch length for most genes in the outgroup *C. lycianthoides*. For example, in the *accD* gene, in addition to *C. lycianthoides*, the species that exhibited highly divergent branches are those belonging to the C_B_ and C_P_ complexes. Furthermore, we also investigated the positive selection of protein-coding genes, and identified 25 putative positive signatures distributed in six out of 79 protein-coding genes (*matK*, *rbcL*, *accD*, *rpl20*, *petD*, and *rpl32*) (Figure 5B).

### 3.4. Chloroplast-Specific Molecular Markers for Capsicum spp.

Comparative analyses allowed us to identify divergent regions potentially useful for an in-depth molecular characterization of the *Capsicum* genus. Among them, we selected those suitable for the development of simple PCR-based molecular markers able to discriminate among different complexes. In Table 2, some examples of three types of potential chloroplast molecular markers in pepper spp. are reported.

Based on SNP variations, the selected coding (*psbA*, *atpI*, *rps2*, *rpoB*, and *atpB*) and non-coding (*rps16* intron) regions discriminated species or complexes through the loss or gain of restriction sites, making them useful for developing cleaved amplified polymorphic sequence (CAPS) markers. In particular, the SNPs present in *psbA* and *atpI* cause the gain and loss of a *Hpy*CH4III and *Hpy*188III restriction sites and were specific to the C_B_ complex; those in the *rps16* intron and *rps2* result in the loss of *EcoR*I and *Sau3*AI restriction sites in *C. chacoense*, and the loss of *Hpy*CH4V and *Alu*I sites in *rpoB* and *atpB* for the C_P_ and C_A_ complexes, respectively.

The *rpl20–rps12* intergenic region showed the highest variability in the SSR, discriminating all three species complexes. By contrast, an SSR detected in the *ycf3* intron is specific to *C. chacoense*, and microsatellites present in the *psbK–psbI* and *atpB–rbcL* intergenic regions univocally characterize *C. pubescens.* The *rpl32–trnL* intergenic region includes an SSR discriminating *C. galapagoense* from the remaining species of the C_A_ complex.

Compared with other potential molecular markers, tandem repeats showed lower discriminatory power among species complexes. In fact, the TRs present in the *ccsA–ndhD* intergenic and *ycf1* coding regions distinguished the C_B_ complex, whereas TRs within the *trnS–rps14* and *ycf2–trnI* regions differentiated *C. pubescens* and species belonging to the C_A_ complex, respectively.

**Figure 6:**
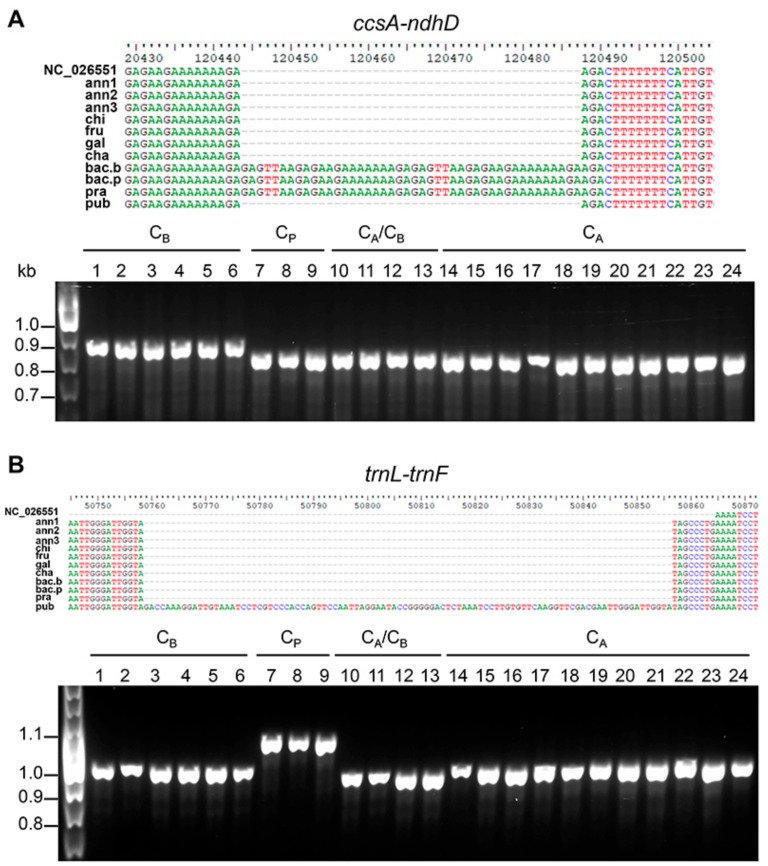
Examples of chloroplast molecular markers developed in this study. PCR markers based on the presence of perfect tandem repeats and insertions/deletions (InDels) able to discriminate C_B_ (**A**) and C_P_ (**B**) complexes. PCR results from representative genotypes in each complex are shown. C_B_ = *C. baccatum*; C_P_ = *C. pubescens*; C_A_ = *C. annuum*; 1 = bac.b; 2 = bac.p; 3 = bac.p2; 4 = bac.p3; 5 = bac.p4; 6 = pra; 7 = pub; 8 = pub2; 9 = pub3; 10 = cha; 11 = cha2; 12 = cha3; 13 = cha4; 14 = ann2; 15 = ann4; 16 = ann5; 17 = ann6; 18 = ann7; 19 = chi2; 20 = chi3; 21 = chi4; 22 = fru2; 23 = fru3; 24 = fru4.

A preliminary experimental validation in representative species confirmed the presence of the tandem repeat detected in the *ccsA–ndhD* intergenic region in genotypes of the C_B_ complex, and the insertion in the *C. pubescens* plastome of a sequence of 98 bp in length in the *trnL–trnF* region that was previously undescribed (Figure 6). The validation of other candidate markers listed in Table 2 is currently underway, and it will be the main objective of a future study.

## 4. Discussion

Until the ‘80s, the sequencing of single chloroplast genes and/or of non-coding regions was widely used for phylogenetic studies with the purpose of clarifying inter- and intra-species relationships and investigating plastid DNA diversity [31,46]. Improvements in protocols for chloroplast isolation and cpDNA extraction, coupled with the evolution and spread of NGS techniques, made complete plastid genome sequencing affordable [32,47]. This promptly allowed the extension of gene-based phylogenetics to phylogenomics and going beyond traditional molecular marker-based barcoding approaches. Indeed, the number of plastid genome sequences released into the public domain for land plant species is growing at an unprecedented rate (see https://www.ncbi.nlm.nih.gov/genome/browse#!/organelles/). Several projects were recently undertaken with the aim of obtaining multiple complete chloroplast genomes and providing basic information for comparative analysis [14,48].

At present, ten cpDNA sequences are available in Genbank for *Capsicum* species. With the present study, we contributed to enriching the cpDNA sequence space available for *Capsicum* by releasing into the public domain the plastomes of eleven genotypes. Based on this resource, we performed the first large-scale genome-wide analysis of molecular diversity of *Capsicum* species belonging to the three main taxonomic complexes. Mapping of reads ranges from 36 to 2581 per-base coverage across chloroplast genomes. Although we recorded a 72-fold difference in mean sequence depth per base among plastomes, this coverage was nevertheless sufficient to assemble all full-length genomes. The plastomes were fairly well conserved in terms of size, gene arrangement, and gene number, and comparable with those of *Capsicum* species available at GenBank. In order to evaluate within-species nucleotide variability, we compared already publically available *Capsicum* plastomes with those produced in this work. Pairwise sequence comparisons showed that sequences belonging to the same species are almost identical, even if a variable number of SNPs and InDels was identified. A subset of the InDel events we observed fall within mono-nucleotide repetitive stretches (mainly A/T), which are known to be prone to sequencing errors. The remaining InDels affect tandem repeats and, to a lesser extent, microsatellite or low-complexity regions. It cannot be excluded that these InDels may be due to errors in the assembly procedure. Indeed, the assembly of large tandem-repeat arrays remains intractable especially if the sequencing technique is based on short reads [49].

Although comparative analysis of genomic sequences, which included *C. lycianthoides* as a reference genome, revealed high sequence similarity among the eleven pepper plastomes, a wide spectrum of variations, ranging from point mutations to small/medium-sized InDels, was observed in 67% of the genes. The differences in the most variable genes (e.g., *accD*, *ndhB*, *rpl20*, *ycf1*, and *ycf2*) were due to InDels. In particular, *accD* and *rpl20* showed the highest variability between species as already observed [24] upon comparing the *C. annuum* plastome with those of other Solanaceae. Compared to previous results [24], we found both intra- and inter-specific variability in the C_A_ complex. In addition, we detected a large insertion in the *trnL–trnF* intergenic region of *C. pubescens*, while *ycf1* and *ycf2* were characterized in most genotypes by various InDels.

Single-nucleotide polymorphisms, when normalized per kb, resulted in a comparable number among the eleven plastomes and mostly localized in intergenic regions as expected, since coding regions are, in general, more conserved than non-coding regions [50]. Simple sequence repeats or microsatellites are locus-specific and multi-allelic markers that were extensively involved in a variety of applications including cultivar identification [51], genetic diversity assessment [52], molecular evolution [53], etc. In the present study, SSRs were mainly localized in intergenic regions and in the LSC. This finding is in agreement with previous results for species belonging to Solanaceae, Poaceae, and Arecaceae [5,30,54,55]; indeed, the low number of SSRs within IRs is due to its duplicative nature that implies copy-correction activity [54]. Mono-(p1), di-(p2), and trinucleotide (p3) SSR types were detected in all genotypes under investigation. In particular, the majority involved p1 SSRs, representing 82–87% of the total, whilst tetranucleotides (p4) were only present in species belonging to the C_A_ complex (1.6% of the total). This pattern of distribution was in accordance with previous results on four cultivated species of the *Capsicum* genus, reporting a frequency of 80% for mononucleotides, while tetranucleotides were the least frequent type [5]. The analysis of tandem repeats revealed that their period size was mostly between nine and 30 nucleotides and only one period sequence was longer than 100 nucleotides.

The strong bootstrap support of the ML tree based on the concatamers of seven plastid regions strengthens that it can represent a reliable phylogenetic framework for the assessment of repetitive element evolution in *Capsicum* species. When this phylogenetic tree was compared with variability derived from SSRs and TRs, the species grouping obtained by the ML analysis reflected the observed variability in repetitive sequences. While the C_A_ complex is relatively homogeneous in terms of variation in SNPs and repeats, *C. chacoense* displays a different pattern of variation compared with genotypes in the C_B_ complex, particularly for SNP and SSR variation.

Both the phylogenetic reconstructions based on the concatamers of seven plastid regions and on complete plastid sequence (Figure 3) correspond well to previous reports on the relationships among *Capsicum* species and complexes. However, the stronger bootstrap support of the latter tree allows the confirmation of the taxonomic placement of some critical species as the inclusion of *C. galapagoense* in the C_A_ complex and of *C. praetermissum* in the C_B_ complex. In particular, the *C. chacoense* genotype examined here can be unequivocally assigned as a sister to the C_B_ complex, accordingly with the results of Walsh and Hoot [20], and ruling out the previous hypothesis by Ince, Karaca, and Onus [19], who postulated *C. chacoense* as a sort of bridge placement between the C_A_ and C_B_ complexes. Nevertheless, *C. chacoense* is the basal species in the clade, including the C_B_ complex; thus, it is expected also to share some plesiomorphic traits with the C_A_ complex.

We identified 25 putative positive signatures distributed in six protein-coding genes. Overall, the genes with higher divergence rate also showed higher positive selection. These genes were involved in different essential functions such as the Calvin cycle (*rbcL*), cytochrome b6f (*petD*), RNA maturation (*matK*), ribosomal proteins (*rpl20*, *rpl32*), and fatty-acid biosynthesis (*accD*). The genes under positive selection may be related to a recent increase in diversification rate following adaptation to novel ecological conditions [56,57]. In particular, as it was also found in other plant lineages, we detected both highly divergent branches and accelerated rates of evolution in the *accD* gene, a plastid-encoded subunit of the acetyl-coenzyme A (CoA) carboxylase [58]. *accD* was found to affect plant fitness and leaf longevity [59,60] and might have been involved in the adaptation to specific ecological niches during *Capsicum* diversification.

The sequence variations identified here were used to develop simple PCR-based markers useful to distinguish species belonging to different complexes. Single-nucleotide polymorphism SNP analysis led us to identify variation in the gain and loss of restriction sites allowing the development of CAPS markers, allowing the discrimination of different complexes. In particular, SNPs present in *psbA* and *atpI* were specific for the C_B_ complex, whereas SNPs in *atpB* allowed discrimination among species belonging to the C_A_ complex. The use of SSRs in intergenic regions as molecular markers was widely suggested, since these regions evolve faster than coding sequences [31,55]. Among them, we identified the variation in the *atpB–rbcL* region, already reported by Walsh and Hoot [20], that elucidated relationships between *Capsicum* spp., thereby contributing to their taxonomic grouping. We selected and tested, in a representative sample of *Capsicum* species, a TR in the *ccsA–ndhD* region that clearly enabled us to discriminate species belonging to the C_B_ complex. Furthermore, in the *trnL–trnF* intergenic region, we identified and tested an insertion of 98 bp in *C. pubescens* different from that of 225 bp found in *C. annuum* by Jo et al. [24]. Moreover, Jarret [61] evaluated the feasibility of using this locus for DNA barcoding within the *C. annuum* complex and demonstrated its ability to differentiate among the examined species. Generally, plant DNA barcoding often showed their limit in species discrimination, especially for closely related taxa, making unrealistic the possibility of using a universal set of markers for species identification in higher plants. This limitation is further exacerbated for application at the intra-specific level. There is an increasing interest in expanding the genetic diversity in the production chain, as well as in the genetic traceability of foods with specific metabolic traits. While traditional barcoding often struggles to reliably differentiate within *Capsicum* complexes, full plastome sequences proved powerful to distinguish each cultivar, by virtue of global patterns of sequence variations. Indeed, thanks to the use of the full plastome barcode as the method of choice for plant identification, we envisage a growing use of full-length plastomes in the identification and traceability of pepper varieties.

## 5. Conclusions

The present study reports the complete plastomes of eleven genotypes belonging to the three main taxonomic species complexes of *Capsicum*. This sequence resource was exploited for the genome-wide analysis of molecular diversity within the *Capsicum* genus. Comparative analysis revealed a wide spectrum of variations, some of which were found at both the inter- and intra-specific level. Our phylogenetic reconstruction corresponds well to previous reports on the relationships among *Capsicum* species and complexes, but contributes to the taxonomic placement of some critical species. In particular, the *C. chacoense* genotype examined here can be unequivocally assigned as a sister to the C_B_ complex. Furthermore, we identified 25 putative positive signatures distributed in six protein-coding genes involved in different essential functions of chloroplasts and probably related to the recent increase in diversification rate following adaptation to novel ecological conditions. Finally, the sequence variations allowed us to develop simple PCR-based markers that can be helpful to distinguish species belonging to different complexes.

## Figures and Tables

**Table 1:** Plastome features of the eleven *Capsicum* genotypes.

Genotype Code	Species	Complex ^a^	Germplasm Bank Identifier (ID)	Size (Base Pairs)				% GC
				Total	LSC ^d^	SSC ^d^	IR ^d^	
ann1	*C. annuum*	C_A_	CGN21526 ^b^	157,052	87,380	17,882	25,895	37.71
ann2	*C. annuum*	C_A_	CAP319 ^c^	156,842	87,380	17,960	25,751	37.72
ann3	*C. annuum*	C_A_	CAP1546 ^c^	156,872	87,341	17,917	25,807	37.73
chi	*C. chinense*	C_A_	CGN22099 ^b^	156,858	87,288	17,860	25,855	37.73
fru	*C. frutescens*	C_A_	CGN22779 ^b^	156,836	87,359	17,911	25,783	37.72
gal	*C. galapagoense*	C_A_	CGN22208 ^b^	157,029	87,366	17,941	25,861	37.69
cha	*C. chacoense*	C_A_/C_B_	CGN22084 ^b^	156,841	87,346	17,893	25,801	37.72
bac.b	*C. baccatum* subsp. *baccatum*	C_B_	CGN23261 ^b^	157,053	87,350	17,973	25,865	36.45
bac.p	*C. baccatum* subsp. *pendulum*	C_B_	CGN21512 ^b^	157,144	87,351	17,973	25,910	37.66
pra	*C. praetermissum*	C_B_	CGN20805 ^b^	157,056	87,351	17,973	25,866	37.66
pub	*C. pubescens*	C_P_	CGN22108 ^b^	157,390	87,688	17,928	25,887	37.69

^a^ Walsh and Hoot [20] and Ince, Karaca, and Onus [19]; C_A_: *C. annuum*; C_B_: *C. baccatum*; C_P_: *C. pubescens*; ^b^ from the Centre for Genetic Resources germplasm bank, The Netherlands; ^c^ from IPK Gatersleben germplasm bank, Germany; ^d^ LSC = large single-copy region; SSC = small single-copy region; IR = inverted repeat; GC = guanine/cytosine.

**Table 2:** Examples of chloroplast molecular markers (single-nucleotide polymorphisms, SNPs; simple sequence repeats, SSRs; tandem repeats, TRs) identified in this study using the accession NC_026551 of *C. lycianthoides* as a reference.

		Genotypes											
Marker	Region	ann1	ann2	ann3	chi	fru	gal	cha	bac.b	bac.p	pra	pub	Notes
*SNP* ^a^													
AAACC[A/G]TTTA	*psbA*	0 ^b^	0	0	0	0	0	0	1	1	1	0	Gain of a *HpyCH4*III restriction site
GAATT[C/A]TATC	*rps16* intron	0	0	0	0	0	0	1	0	0	0	0	Loss of a *EcoR*I restriction site
ATATT[C/T]CCGA	*atpI*	0	0	0	0	0	0	0	1	1	1	0	Loss of a *Hpy188*III restriction site
TGCGA[G/T]ATCG	*rps2*	0	0	0	0	0	0	1	0	0	0	0	Loss of a *Sau3A*I restriction site
TCTTG[C/A]ATAT	*rpoB*	0	0	0	0	0	0	0	0	0	0	1	Loss of a *HpyCH4*V restriction site
CCAGC[T/C]CCCC	*atpB*	1	1	1	1	1	1	0	0	0	0	0	Loss of a *Alu*I restriction site
*SSR* ^c^													
TTTC(A)_n_TCAT	*psbK–psbI*	9 ^d^	9	9	9	9	9	10	10	10	10	2	
TCTG(T)_n_CAAA	*trnG–trnR*	12	12	12	12	12	12	11	11	11	11	10	
AAT(ATAA)_n_AT	*psaA–ycf3*	4	4	4	4	4	4	3	2	2	2	3	
CTTC(CT)_n_TATC	*ycf3* intron	5	5	5	5	5	5	4	5	5	5	5	
TTTC(A)_n_GGTA	*atpB–rbcL*	11	11	11	11	11	11	9	9	9	9	8	
GTTA(T)_n_AGGT	*rpl20–rps12*	14	14	14	14	14	14	15	16	16	16	13	
TAAC(T)_n_GTTG	*rpl32–trnL*	6	6	6	6	6	9	6	6	6	6	6	
*TR* ^e^													
GGAT(TTATC…GCCTA)_37_AAGG	*trnS–rps4*	1 ^f^	1	1	1	1	1	1	1	1	1	2	
AAGA(GAGTT…AAAGA)_22_AGAC	*ccsA–ndhD*	1	1	1	1	1	1	1	3	3	3	1	
TTAA(TTGGT…TTGTT)_30_TAAG	*ycf1*	1	1	1	1	1	1	1	2	2	2	1	
TCTC(ATTGA…ATTGT)_25_ATTT	*ycf2–trnI*	2	2	2	2	2	2	1	1	1	1	1	

^a^ The nucleotide in brackets (underlined) represents the alternative allele; ^b^ 0 = reference allele; 1 = alternative allele; ^c^ the nucleotide(s) in parentheses represents the repeat unit; n = number of repeats; ^d^ different numbers correspond to the number of repeat unit in each genotype; ^e^ the nucleotides in parentheses represent the tandem repeat, the number out of parentheses corresponds to the length of repeat; ^f^ different numbers correspond to the number of tandem repeats in each genotype.

## Supplementary Materials

The following are available online at http://www.mdpi.com/2073-4425/9/10/503/s1, Figure S1: Comparison of plastome junctions (LSC/IRb, SSC/IRa, IRb/SSC, and IRa/LSC) among pepper species. Numbers indicate the lengths of intergenic spacers (IGSs), genes, and spacers around IR/LSC and IR/SSC junctions; Figure S2: Comparison of eleven Capsicum plastome sequences using the VISTA software and the accession NC_026551 of *C. lycianthoides* as a reference. Blue and red regions correspond to coding and non-coding regions, respectively. The *Y*-axis represents percent similarity ranging from 50–100%; Figure S3: Distribution of single-nucleotide polymorphisms (SNPs) in the eleven *Capsicum* plastomes using the accession NC_026551 of *C. lycianthoides* as a reference. Number and SNP distribution among different regions: exon, intron, intergenic region, large single-copy region (LSC), small single-copy region (SSC), and inverted repeat b (IRb). The number of SNPs (left bar) does not correspond to SNP distribution (right bar) due to overlap of several genes on opposite strands; Figure S4: Distribution of simple sequence repeats (SSRs) in the eleven *Capsicum* plastomes and in the accession NC_026551 of *C. lycianthoides* used as an outgroup species. (A) Total number of SSRs reported as SSR type. (B) Number and SSR distribution among different regions: exon, intron, intergenic region, large single-copy region (LSC), small single-copy region (SSC), and inverted repeat b (IRb). P1 = mono-, p2 = di-, p3 = tri-, p4 = tetranucleotide; Figure S5: Distribution of perfect tandem repeats (TRs) in the eleven *Capsicum* plastomes and in the accession NC_026551 of *C. lycianthoides* used as an outgroup species. TR distribution among different regions: exon, intergenic region, large single-copy region (LSC), small single-copy region (SSC), and inverted repeat b (IRb); Figure S6: Molecular phylogenetic analysis using maximum-likelihood method and SNP variation across exon, intron, and intergenic regions among eleven *Capsicum* plastomes. (A) Phylogenetic tree inferred from maximum-likelihood analysis of seven combined plastid regions (RAxML maximum-likelihood bootstrap above nodes). Heat maps represent SNP variability in (B) exon, (C) intron, and (D) intergenic regions compared with the *C. lycianthoides* plastome (NC_026551) used as a reference. Yellow corresponds to the reference allele; red and blue correspond to alternative alleles. The arrows indicate the anticlockwise genome orientation; Figure S7: Molecular phylogenetic analysis using maximum-likelihood method and SSR and TR size variation among eleven *Capsicum* plastomes. (A) Phylogenetic tree inferred from maximum-likelihood analysis of seven combined plastid regions (RAxML maximum-likelihood bootstrap above nodes). Heat maps represent differences in SSR size (B) and in the number of copies of perfect tandem repeats (C) compared with *C. lycianthoides* plastome (NC_026551) used as a reference. Heat map colors range from green through yellow to red, where green and red indicate an SSR size greater or lesser than the reference, and a higher and lower number of copies than the reference. The arrows indicate the anticlockwise genome orientation; Table S1: Single-nucleotide polymorphisms (SNPs) in the eleven *Capsicum* plastomes identified using the SNP-sites tool [41] using the accession NC_026551 of *C. lycianthoides* as a reference. “0” indicates the reference allele, “1” or “2” indicates the alternative allele; Table S2: Simple sequence repeats (SSRs) in the eleven *Capsicum* plastomes using the accession NC_026551 of *C. lycianthoides* as a reference. SSR size, location, and distribution among different regions: exon, intron, and intergenic regions are reported. SSRs were identified using the MISA (MIcroSAtellite) identification tool (http://pgrc.ipk-gatersleben.de/misa/); Table S3: Perfect tandem repeats (TRs) in the eleven *Capsicum* plastomes using the accession NC_026551 of *C. lycianthoides* as reference. TR period size, copy number, location, and distribution among different regions: exon, intron, and intergenic regions are reported. TRs were identified using the Tandem Repeats Finder tool (https://tandem.bu.edu/trf/trf.basic.submit.html); Table S4: Summary of within-species nucleotide variability assessed starting from pairwise global alignments.
