# Supplementary material for: The Complete Plastome Sequences of Eleven Capsicum Genotypes: Insights into DNA Variation and Molecular Evolution
Source: Genes (Basel). 2018 Oct 17;9(10):503. doi: 10.3390/genes9100503 (PMC6210379; doi:10.3390/genes9100503)
Supplement: Supplementary file 1 [file genes-09-00503-s001.zip › Suppl mat/Suppl_Figures_DAgostino_et_al_.docx]

Supplementary Figures

The complete plastome sequences of eleven *Capsicum* genotypes: insights into DNA variation and molecular evolution

**Nunzio D’Agostino^1*^, Rachele Tamburino^2^, Concita Cantarella^1^, Valentina De Carluccio^1,3^, Lorenza Sannino^2^, Salvatore Cozzolino^3^, Teodoro Cardi^1^, Nunzia Scotti^2*^**

^1^ CREA Research Centre for Vegetable and Ornamental Crops, Via dei Cavalleggeri 25, 84098 Pontecagnano Faiano (SA), Italy

^2^ CNR-IBBR, National Research Council of Italy, Institute of Biosciences and BioResources, Via Università 133, 80055 Portici (NA), Italy

^3^ Department of Biology, University of Naples Federico II, Via Cinthia, 80126 Naples, Italy

nunzio.dagostino@crea.gov.it (N.DA.); rachele.tamburino@gmail.com (R.T.); concita.cantarella@gmail.com (C.C.); valentina.decarluccio@gmail.com (V.DC.); lorenza.sannino@ibbr.cnr.it (L.S.); cozzolin@unina.it (S.C.); teodoro.cardi@crea.gov.it (T.C.); nscotti@unina.it (N.S.)

***** Correspondence: nscotti@unina.it; Tel.: +39 081 2539482; nunzio.dagostino@crea.gov.it; Tel.: +39 089 386243


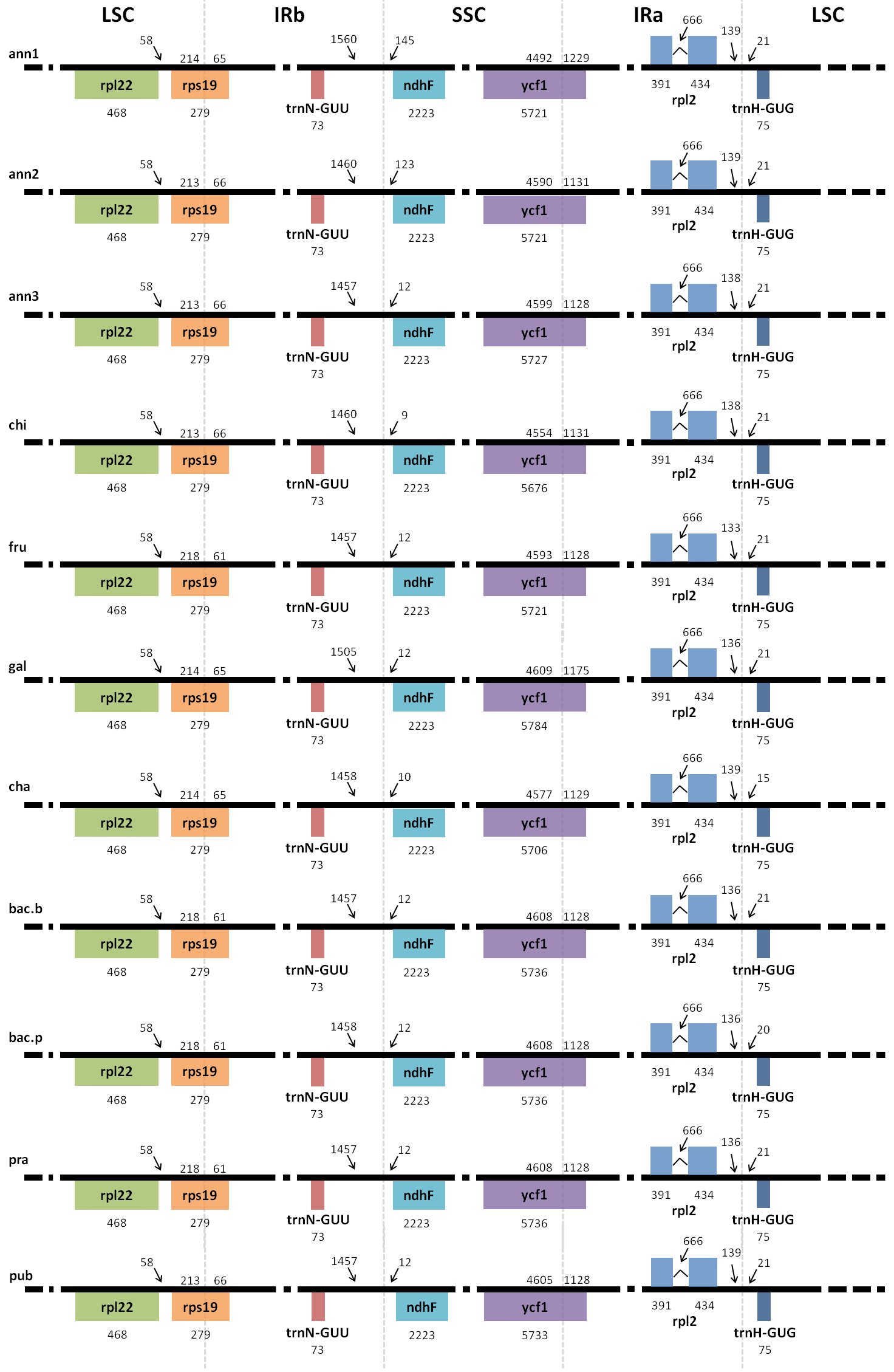


**Figure S1.** Comparison of plastome junctions (LSC/IRb, SSC/IRa, IRb/SSC and IRa/LSC) among pepper species. Numbers indicate the lengths of IGSs, genes, and spacers around IR-LSC and IR-SSC junctions.


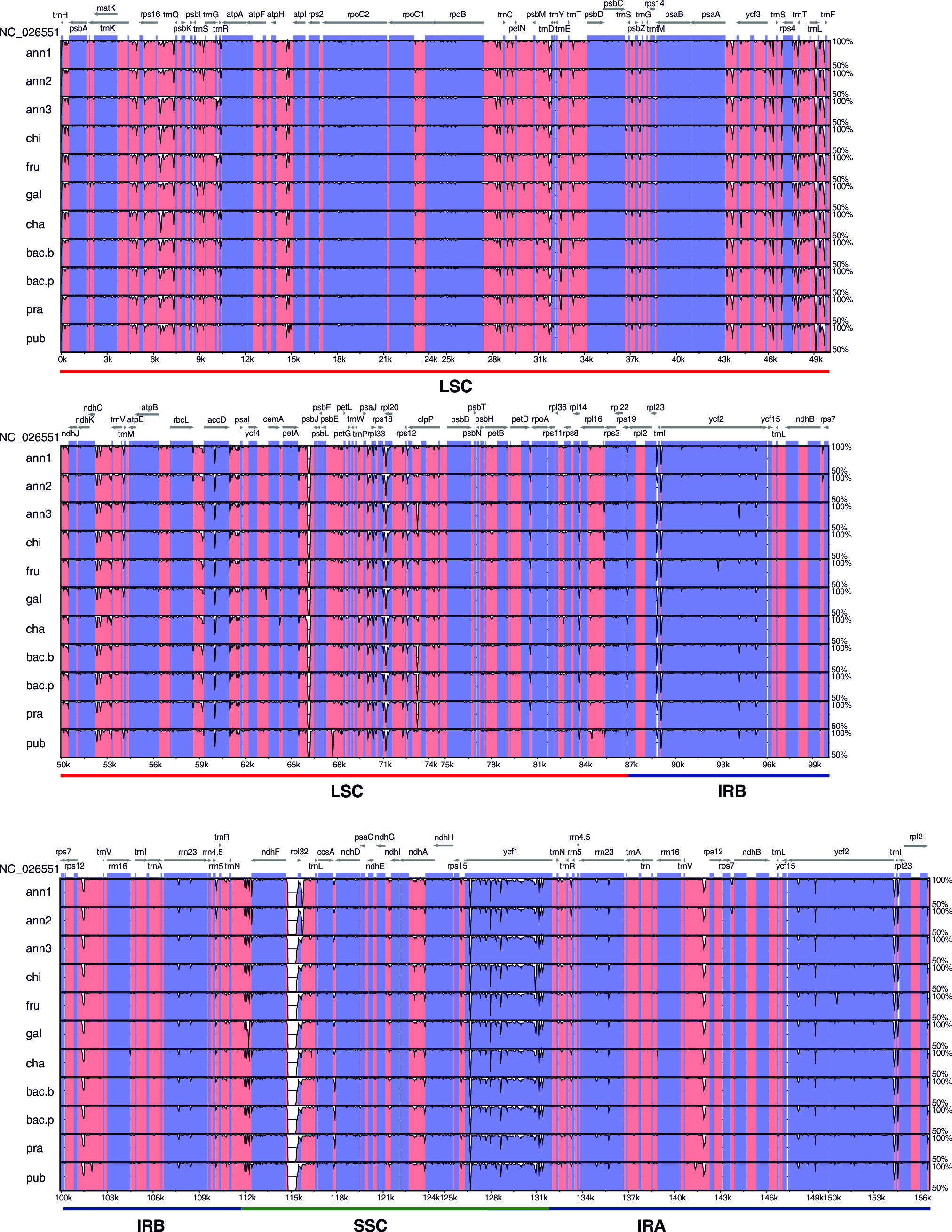


**Figure S2.** Comparison of eleven *Capsicum* plastome sequences using the VISTA software and the accession NC_026551 of *C. lycianthoides* as reference. Blue and red regions correspond to coding and non coding regions, respectively. The Y scale represents % of similarity ranging from 50 to 100%.


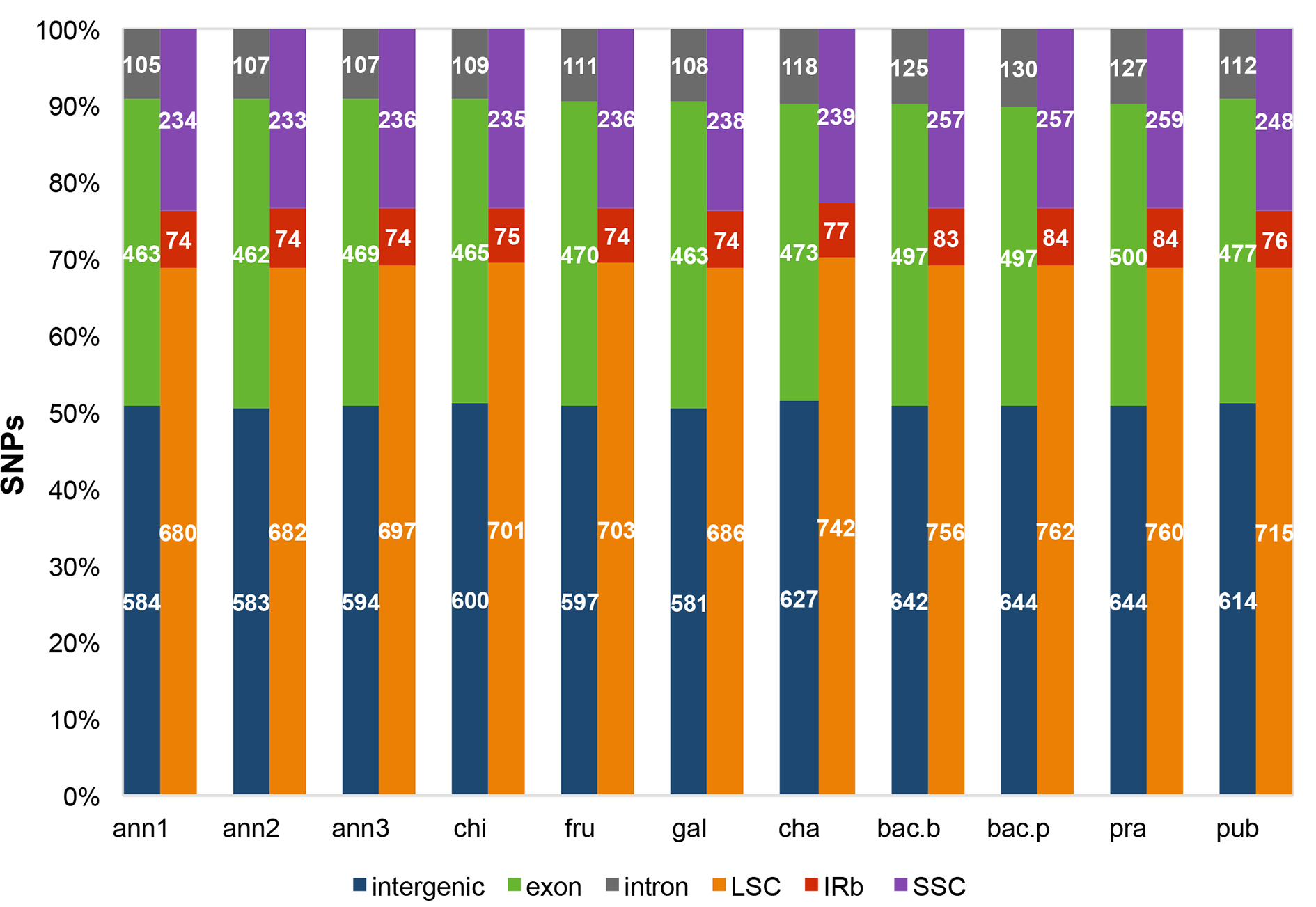


**Figure S3.** Distribution of Single Nucleotide Polymorphisms (SNPs) in the eleven *Capsicum* plastomes using the accession NC_026551 of *C. lycianthoides* as reference. Number and SNP distribution among different regions: exon, intron, intergenic region, Large Single Copy region (LSC), Small Single Copy region (SSC) and Inverted Repeat b (IRb). The number of SNPs (left bar) does not correspond to SNPs distribution (right bar) due to overlap of several genes on opposite strands.

**A**


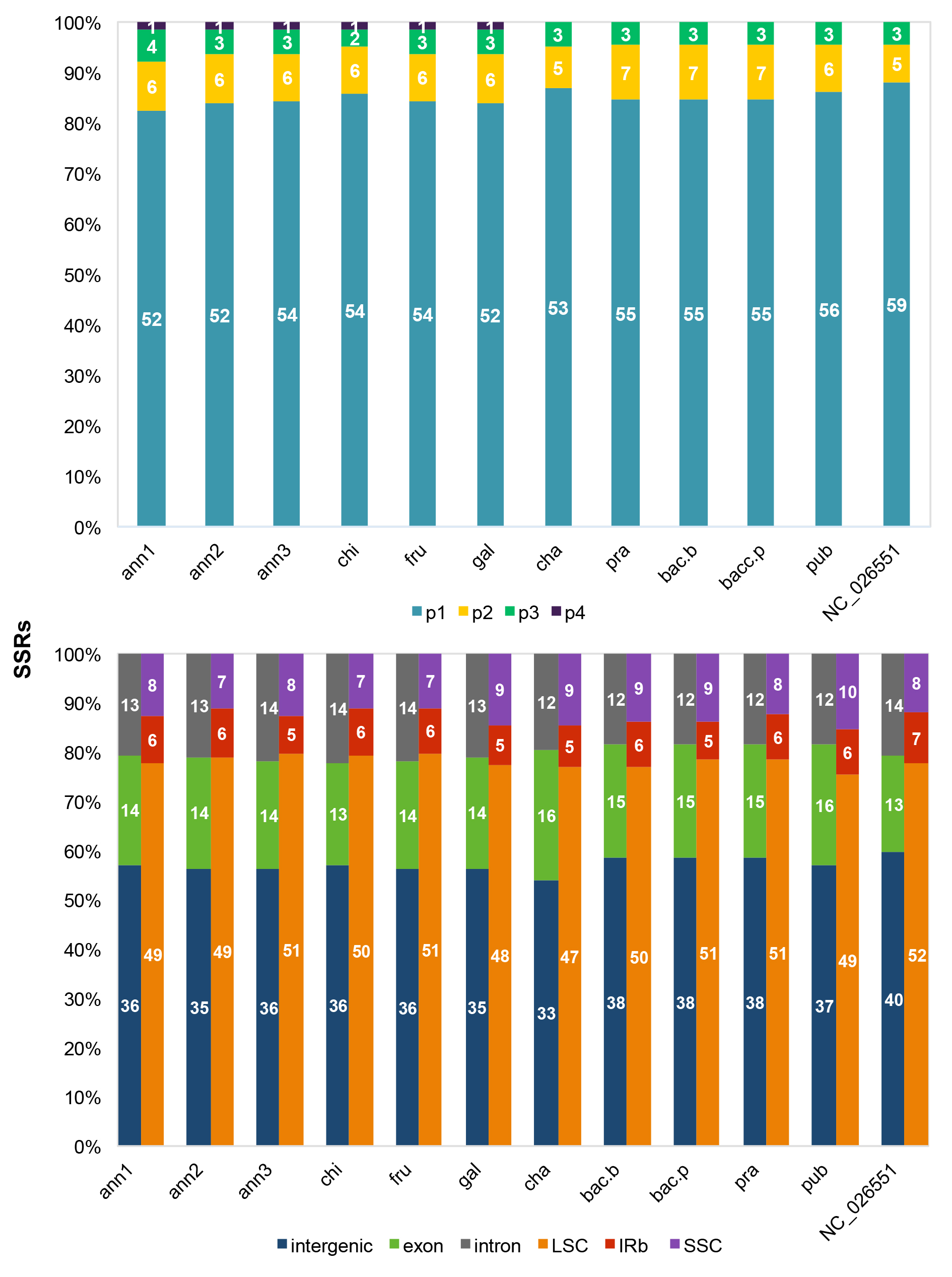


**B**

**Figure S4.** Distribution of Simple Sequence Repeats (SSRs) in the eleven *Capsicum* plastomes and in the accession NC_026551 of *C. lycianthoides* used as outgroup species. (**A**) Total number of SSRs reported as SSR type. (**B**) Number and SSR distribution among different regions: exon, intron, intergenic region, Large Single Copy region (LSC), Small Single Copy region (SSC) and Inverted Repeat b (IRb). P1 = mono-, p2 = di-, p3 = tri-, p4 = tetra-nucleotide.


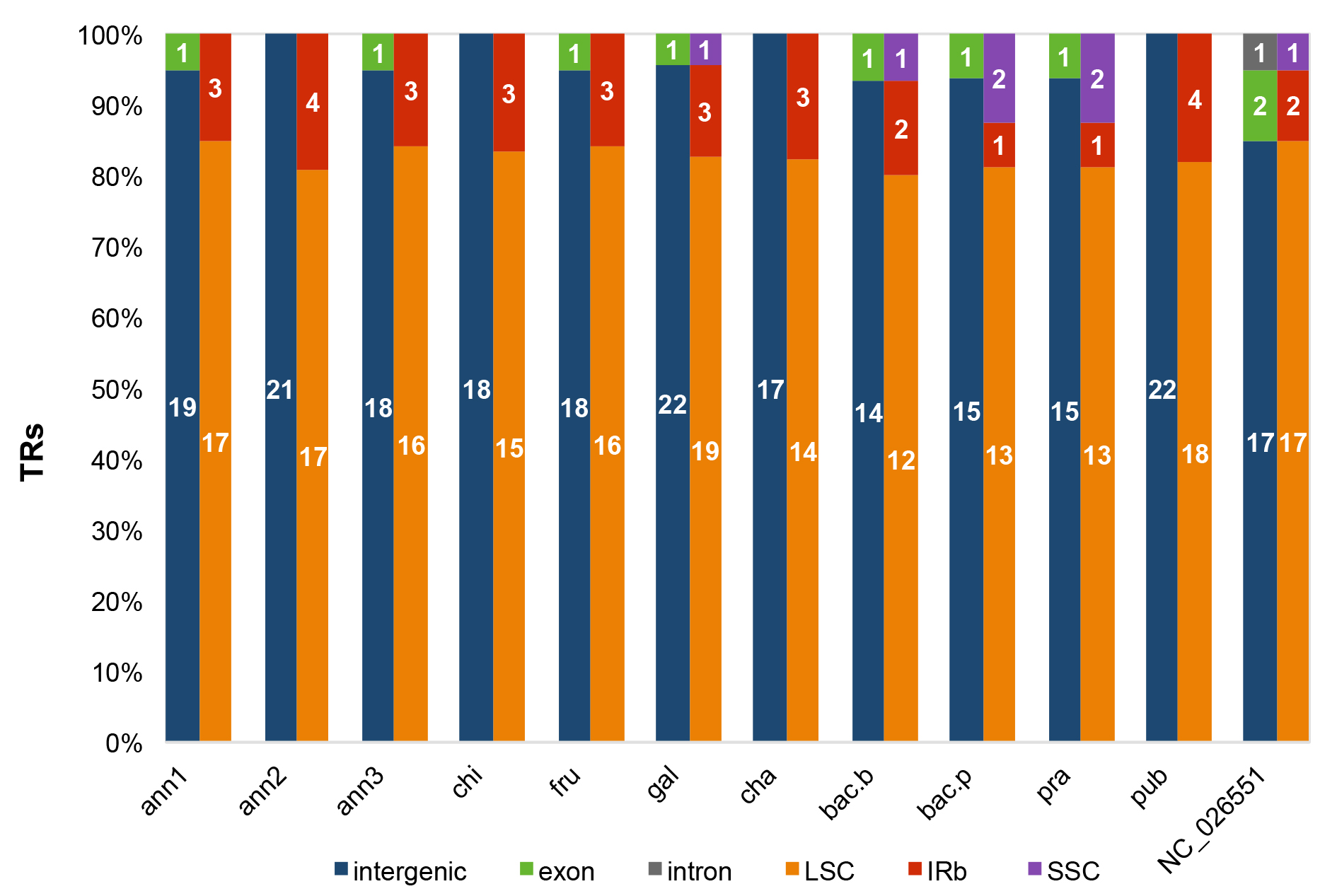


**Figure S5.** Distribution of perfect tandem repeats (TRs) in the eleven *Capsicum* plastomes and in the accession NC_026551 of *C. lycianthoides* used as outgroup species. TRs distribution among different regions: exon, intergenic region, Large Single Copy region (LSC), Small Single Copy region (SSC) and Inverted Repeat b (IRb).


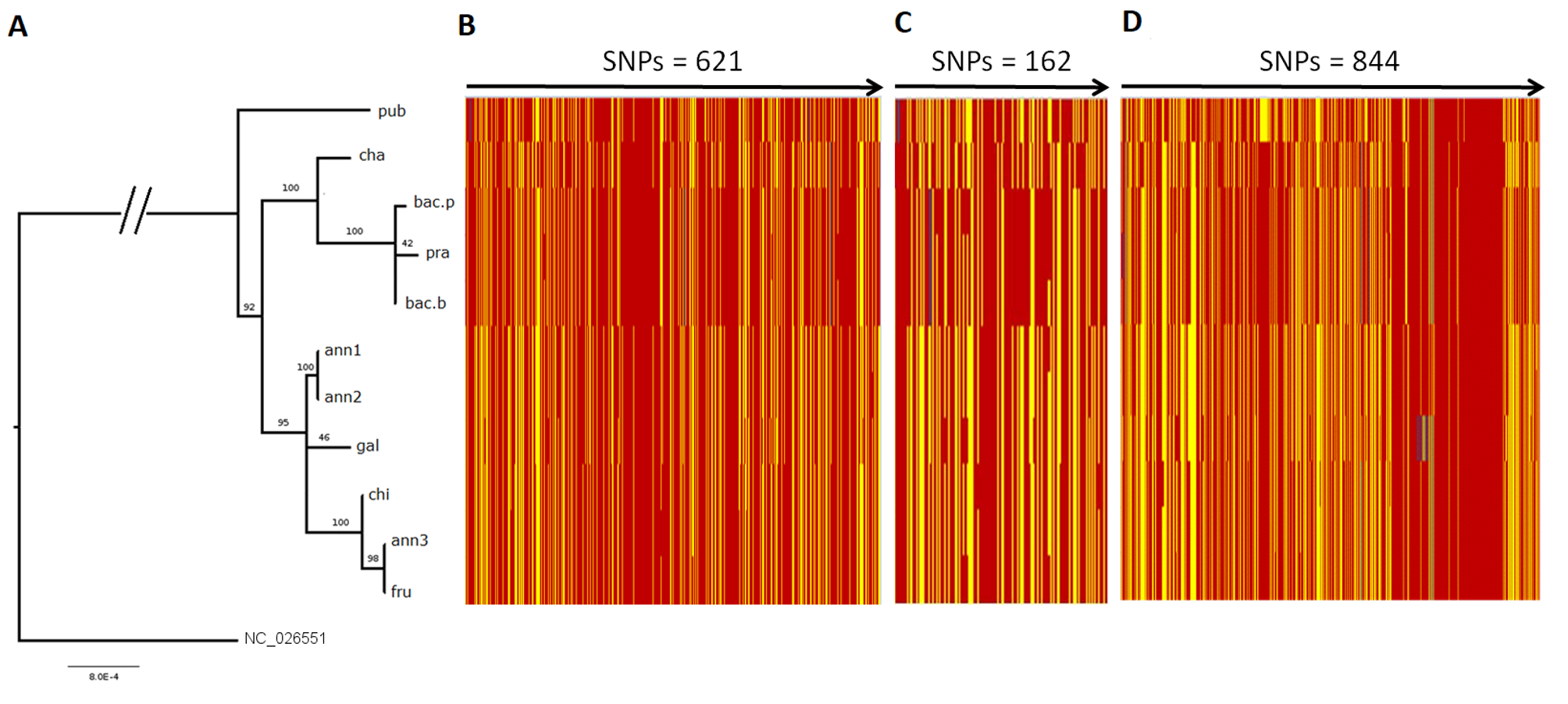


**Figure S6.** Molecular phylogenetic analysis by Maximum Likelihood method and SNP variation across exon, intron and intergenic regions among eleven *Capsicum* plastomes. (**A**) Phylogenetic tree inferred from maximum likelihood analysis of seven combined plastid regions (RAxML maximum likelihood bootstrap above nodes). Heat maps represent SNP variability in (**B**) exon, (**C**) intron and (**D**) intergenic regions compared with the *C. lycianthoides* plastome (NC_026551) used as reference. Yellow corresponds to reference allele; red and blue correspond to alternative alleles. The arrows indicate the anticlockwise genome orientation.


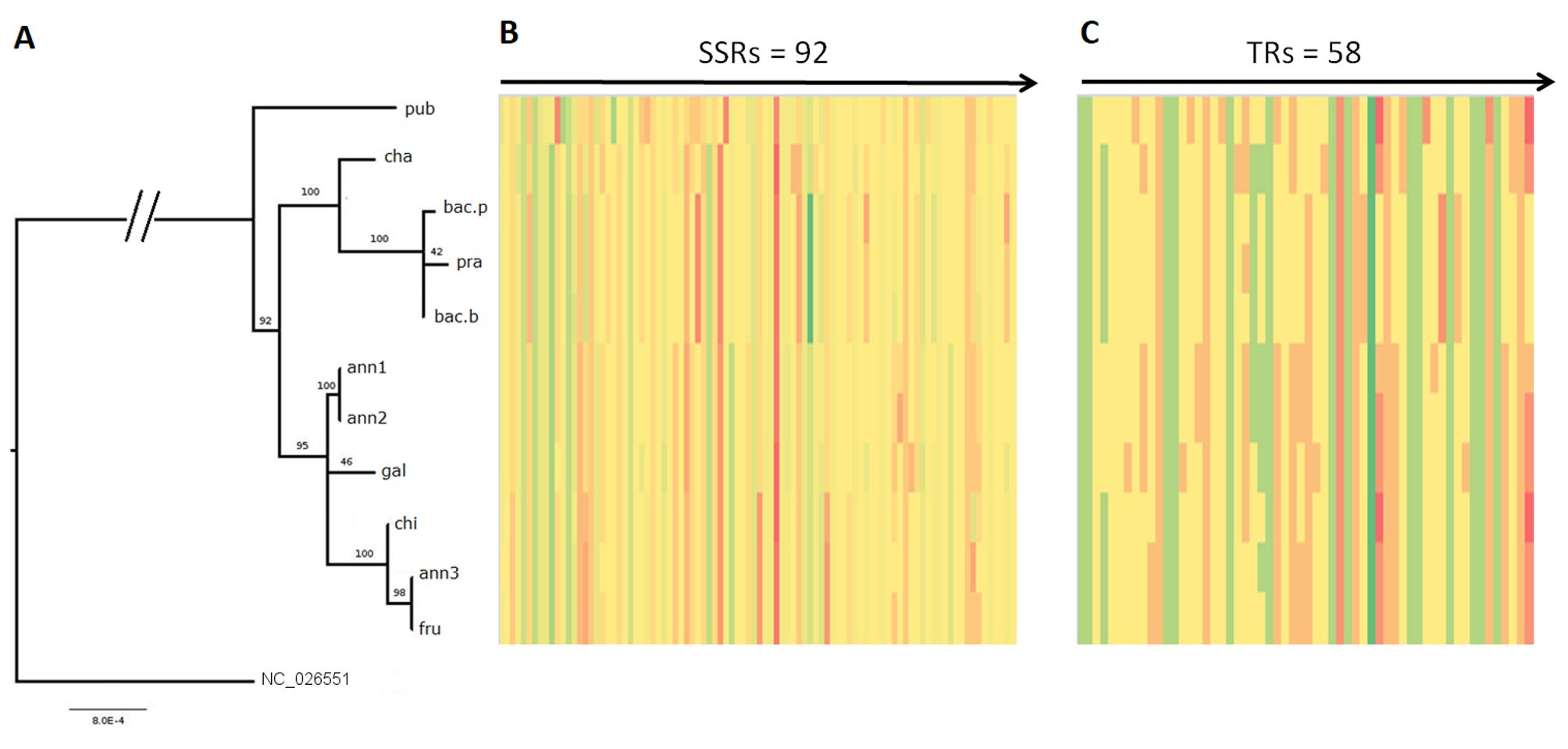


**Figure S7.** Molecular phylogenetic analysis by Maximum Likelihood method and SSR and TR size variation among eleven *Capsicum* plastomes. (**A**) Phylogenetic tree inferred from maximum likelihood analysis of seven combined plastid regions (RAxML maximum likelihood bootstrap above nodes). Heat map represent differences in SSR size (**B**) and in the number of copies of perfect tandem repeats (**C**) compared with *Capsicum* *lycianthoides* plastome (NC_026551) used as reference. Heat map colours range from green through yellow to red, where green and red indicate a SSR size greater or lesser than reference and a higher and lower number of copies than reference. The arrows indicate the anticlockwise genome orientation.
